# A Novel Operative Procedure for Pelvic Organ Prolapse Utilizing a MRI-Visible Mesh Implant: Safety and Outcome of Modified Laparoscopic Bilateral Sacropexy

**DOI:** 10.1155/2015/860784

**Published:** 2015-04-19

**Authors:** Ralf Joukhadar, Gabriele Meyberg-Solomayer, Amr Hamza, Julia Radosa, Werner Bader, Dimitri Barski, Fakher Ismaeel, Guenther Schneider, Erich Solomayer, Sascha Baum

**Affiliations:** ^1^Department of Obstetrics and Gynecology, University Hospital Homburg, Kirrberger Straße 100, 66424 Homburg, Germany; ^2^Department of Obstetrics and Gynecology, Klinikum Bielefeld, Teutoburger Straße 50, 33604 Bielefeld, Germany; ^3^Department of Urology, Lukaskrankenhaus GmbH, Preußenstraße 84, 41464 Neuss, Germany; ^4^Department of Gynecology, University Hospital Charité, Campus Virchow-Klinikum (CVK), Mittelallee 9, 13353 Berlin, Germany; ^5^Department of Radiology, University Hospital Homburg, Kirrberger Straße 100, 66424 Homburg, Germany

## Abstract

*Introduction*. Sacropexy is a generally applied treatment of prolapse, yet there are known possible complications of it. An essential need exists for better alloplastic materials. *Methods*. Between April 2013 and June 2014, we performed a modified laparoscopic bilateral sacropexy (MLBS) in 10 patients using a MRI-visible PVDF mesh implant. Selected patients had prolapse POP-Q stages II-III and concomitant OAB. We studied surgery-related morbidity, anatomical and functional outcome, and mesh-visibility in MRI. Mean follow-up was 7.4 months. *Results*. Concomitant colporrhaphy was conducted in 1/10 patients. Anatomical success was defined as POP-Q stage 0-I. Apical success rate was 100% and remained stable. A recurrent cystocele was seen in 1/10 patients during follow-up without need for intervention. Out of 6 (6/10) patients with preoperative SUI, 5/6 were healed and 1/6 persisted. De-novo SUI was seen in 1/10 patients. Complications requiring a relaparoscopy were seen in 2/10 patients. 8/10 patients with OAB were relieved postoperatively. The first in-human magnetic resonance visualization of a prolapse mesh implant was performed and showed good quality of visualization. *Conclusion*. MLBS is a feasible and safe procedure with favorable anatomical and functional outcome and good concomitant healing rates of SUI and OAB. Prospective data and larger samples are required.

## 1. Introduction

Surgical treatment of pelvic organ prolapse (POP) underwent a remarkable transformation over the last decade. Starting with facilitated use of vaginal meshes through simplified mesh kits and followed by Food and Drug Administration (FDA) warnings about their safety there has been a change in practice patterns among urogynecologists. One of the observed trends seems to be a decrease of vaginal mesh use and an increase in sacropexy [1–3].

Abdominal sacropexy which represents the “gold standard” in POP surgery is associated with apical success rates of 93–99% along with low recurrence rates [4]. The laparoscopic sacropexy seems to achieve similar success rates in addition to having advantages of less blood loss, reduced morbidity, and shorter hospital stay [5].

Nevertheless it seems that postoperative dysfunction may have a negative effect upon patient's satisfaction. New onset bowel (10–50%), voiding (18%), and sexual (8%) dysfunction after sacropexy have been described [6–8]. In current literature reports on de-novo stress incontinence after sacropexy, as well as on the obstructed defecation syndrome, are to be found [9, 10]. A further possible complication after sacropexy is mesh erosion. In some recent publications the rates of mesh erosion after sacropexy showed up to be unexpectedly high [11].

Thus there is a challenge to optimize this procedure by site specific defect repair to obtain a better anatomic reconstruction.

Furthermore, the chosen alloplastic material according to its biomechanical characteristics may play a role in minimizing mesh-related complications. There are some data available about nonpolypropylene meshes [12, 13]. In an effort to increase patient's safety some of these meshes have been developed to be MRI-visible [14].

The objective of this study was, therefore, to investigate the safety and outcome of a modification of laparoscopic sacropexy in an effort to abate postoperative complications and dysfunction. In this procedure we utilized a MRI-visible mesh implant with good biomechanical characteristics.

## 2. Material and Methods

We report on patients who underwent modified laparoscopic bilateral sacropexy (MLBS) between April 2013 and June 2014.

The selected patients consisted of women with symptomatic uterine or vault prolapse ICS POP-Q stages II or III along with overactive bladder OAB symptoms. The OAB was diagnosed either by urodynamic, micturition diary, or both. Patients with previous vault prolapse surgery of any kind and those with contraindications for sacropexy were excluded.

In patients with previous hysterectomy we performed a sacrocolpopexy and in those without previous hysterectomy we performed a laparoscopic supracervical hysterectomy along with a sacrocervicopexy. In the latter a negative pap-smear no older than 6 months was required preoperatively.

Out of 32 patients that fulfilled the mentioned criteria and who were eligible for sacropexy in terms of adherence to the guidelines only 10 patients decided to undergo MLBS after obtaining informed consent.

To ensure patients' safety we conducted a very strict and frequent follow-up program. Patients were invited to the follow-up at 1, 3, 6, and 12 months. The follow-up took place at the urogynecology department and included a gynecologic examination, a POP-Q determination, and evaluation of micturition diaries.

All patients signed up an informed consent giving permission to use their medical data.

### 2.1. Surgical Procedure

#### 2.1.1. Intra-Operative Setting

The procedure is performed under general anesthesia in the dorsal lithotomy position. A 14-F catheter is inserted into the bladder and a uterus or vaginal manipulator is placed transvaginally.

After establishing a CO_2_-pneumoperitoneum a 10 mm transumbilical trocar is used for the laparoscopy. Two additional 5 mm access ports are placed medial to and 3 cm superior to the anterior superior iliac spine laterally to the epigastric vessels on each side. One 12 mm access port (12 mm Versaport) is placed 3 cm superior to the symphysis pubis.

#### 2.1.2. Dissection of the Lower Point of Mesh Attachment

In patients with previous hysterectomy we performed a dissection of the vaginal stump and in those without previous hysterectomy we performed a laparoscopic supracervical hysterectomy.

#### 2.1.3. Dissection of a Tunnel for Mesh Placement along the Lateral Pelvic Wall at Each Side

In order to perform a site specific repair of the impaired uterine suspension dissection of a peritoneal tunnel for later mesh placement through the superficial portion of the uterosacral ligament (USL_s_) is undertaken.

Identifying the USL is facilitated by ventral traction at the cervical stump or the vault via vaginal manipulator and simultaneous lifting of the rectum cranially and to the contralateral side. After the peritoneal fold overlying the USL_s_ is depicted blunt dissection of a subperitoneal tunnel is performed using a 5 mm overholt-clamp utilized through the contralateral lower access port.

The preparation is started on the right side and is performed strictly subperitoneally to avoid injury to nearby ureter or parts of the inferior hypogastric plexus (IHP).

The sacral end of the created tunnel corresponds to the upper insertion point of the uterosacral ligaments (USL). This point lays on the anterior surface of the sacrum 3 cm caudal to the promontory and 1.5 cm lateral to the midline. Having identified this point of insertion, the overlying peritoneum is incised and the underlying tissue is bluntly dissected: iliac vessels are bluntly pushed laterally and tissue containing parts of the IHP is pushed medially, thus revealing the periosteum of the anterior surface of S2.

**Figure 1:**
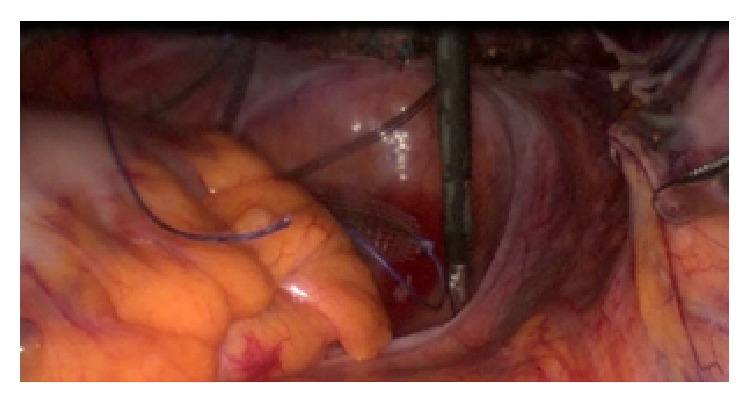


On the left side the procedure is performed identically. It is important to mention that the preparation at the left side is far more difficult due to the anatomical lay of the rectosigmoid junction that must be sufficiently mobilized (Figure 1).

#### 2.1.4. Lower Mesh Fixation/Fixation on the Descent Part

The mesh is inserted via the 12 mm port. The utilized mesh is knitted from nonabsorbable, biostable polyvinylidene fluoride (PVDF) monofilament. We used one of two meshes (DynaMesh-CESA for sacrocervicopexy and DynaMesh-VASA for sacrocolpopexy). Each consists of two thin mesh arms to be placed alongside the lateral pelvic wall with broad ends to be used for sacral fixation and a central part to be attached to the cervical stump or vaginal vault.

A nonabsorbable suture (2.0 Ethibond) is used to fix the central part of the mesh by four simple interrupted sutures.

**Figure 2:**
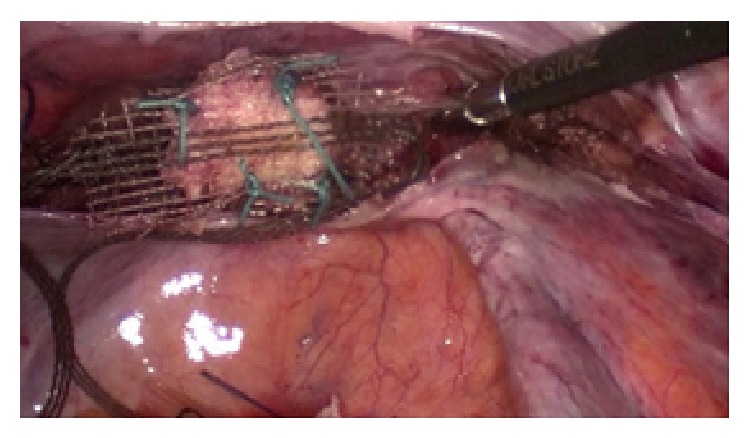


In case of sewing the mesh to the vaginal vault the same suture can be used but attention should be paid to prevent penetrating the full thickness of the vaginal wall (Figure 2).

#### 2.1.5. Bilateral Mesh Placement through the Created Tunnels

**Figure 3:**
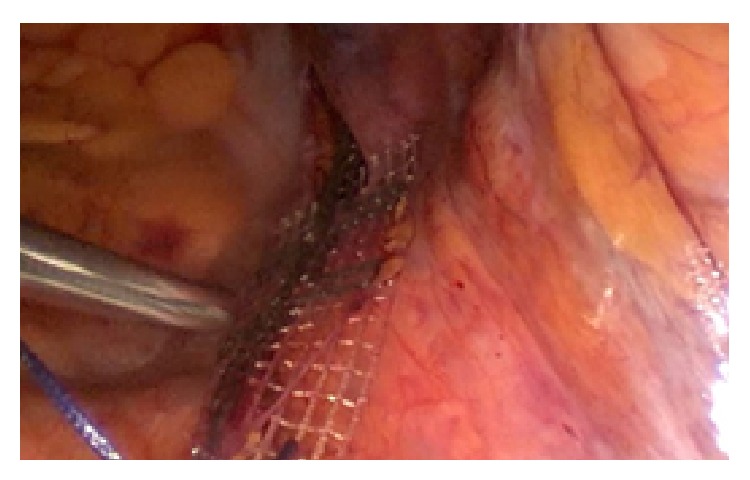


Now the tip of the thin right mesh arm is pulled through the formerly created tunnel. The same is done on the left side, thus achieving a reinforcement of the USL_s_ (Figure 3).

#### 2.1.6. Sacral Fixation at the Level of the Upper Boarder of S2

**Figure 4:**
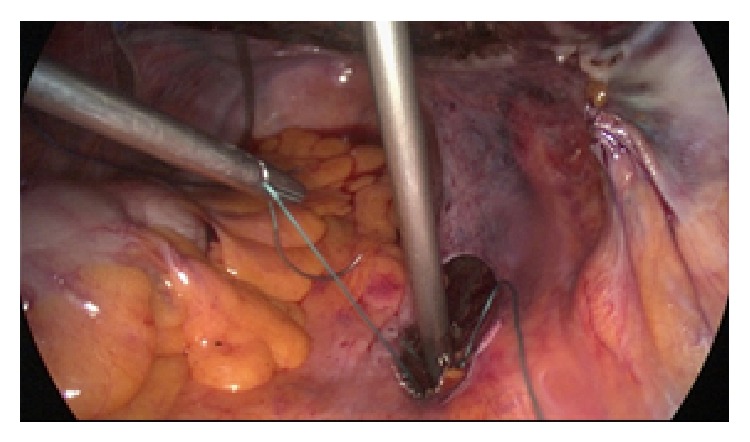


Two interrupted nonabsorbable sutures (2.0 Ethibond) are used for the fixation of the broad end of the lateral mesh arm to the periosteum of the anterior surface of S2 at each side (Figure 4).

#### 2.1.7. Peritoneal Closure

Closure of the peritoneum is achieved via a continuous suture with an absorbable suture (3.0 Vicryl).

### 2.2. Visualizing the Mesh in Magnetic Resonance Imaging (MRI)

Imaging was performed on a 1.5 Tesla Magnet (Magnetom-Aera Siemens, Erlangen, Germany) using a body-array surface coil, placed over the pelvis.

Imaging protocol included both 3D and 2D T1-weighted and T2-weighted sequences. For optimal depiction of the implants, coronal minimum-intensity projections of the 3D datasets were performed.

## 3. Results

We had performed the MLBS on 10 patients between April 2013 and June 2014. All the operations were performed by the same surgeon at an academic university hospital in Germany.

Preoperative risk factors and morbidity data were analyzed.  Table 1 shows the baseline characteristics. We noticed that all patients had given birth via spontaneous vaginal delivery and that 6 (6/10) of them have had 3 or more deliveries. Furthermore, 2 (2/10) of the patients have had a macrosomic baby weighing ≥ 4,500 grams.

In terms of previous prolapse surgery, 2 (2/10) patients have had previous anterior colporrhaphy and 1 (1/10) patient has had concomitant anterior and posterior colporrhaphy for two times in her past medical history.

Regarding the prolapse, 4 (4/10) patients were POP-Q stages II and 6 (6/10) POP-Q stage III preoperatively (Table 2). We took the mean of each POP-Q measurement in all patients to estimate the POP-Q stage resulting from the descent of each compartment by its own (Table 3) to facilitate later comparison with the postoperative results.

All selected patients were suffering from urgency and frequency, of whom 9 (9/10) suffered from OAB-dry and 1 (1/10) from OAB-wet. Further 6 (6/10) patients were suffering from stress urinary incontinence (SUI). The mean frequency of micturition was 13.3 mic./d. and the mean nocturia was 2.3 micturitions/night (Table 4).

Table 5 shows the perioperative data. Low blood loss is reflected by rather small change in hemoglobin levels postoperatively. Regarding concomitant operations, only 1 (1/10) patient required an anterior and posterior colporrhaphy. In the other 9 (9/10) patients the anterior and posterior compartments were sufficiently corrected after performing the MLBS.

Anatomical success was defined as POP-Q stage 0 or I. Postoperative results show that 2 (2/10) patients were POP-Q stage 0 and 8 (8/10) were POP-Q stage I.

Using the mean of each POP-Q measurement in all patients to estimate the POP-Q stage of each compartment by its own shows a mean postoperative POP-Q stage 0 for the middle and posterior compartments and a POP-Q stage I for the anterior compartment (Table 3).

**Figure 5:**
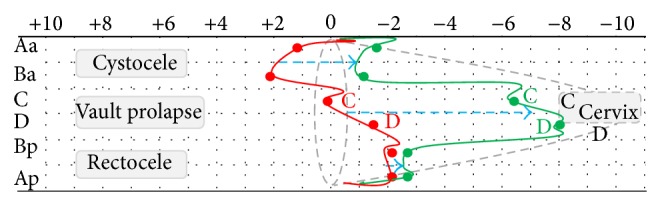
Correction of prolapse in each compartment. The dots correlate to the mean measurements of Aa, Ba, C, D, Ap, and Bp. Red line: lines out the preoperative status (POP-Q measurements). Green line: lines out the postoperative status (POP-Q measurements) before discharge.

The difference between the pre- and postoperative status is lined out in Figure 5.

Postoperative evaluation of urinary incontinence showed that 8 (8/10) patients did not suffer from OAB anymore, whereas 1 (1/10) patient had persistent OAB-dry. One other patient (1/10) had a reduction of her urgency and frequency that was reduced postoperatively from 11 to 7-8 mic./d. Yet she reported bothersome mild urgency despite absent proof of OAB. We regarded that as persistent OAB (Table 4).

Out of 6 (6/10) patients with preoperative SUI only 1 (1/6) patient had persistent SUI postoperatively. Additionally 1 (1/10) patient was diagnosed with de-novo SUI. The mean frequency of micturition was reduced to 8 mic./d. and the mean nocturia to 1.2 micturitions/night (Table 4).

Table 6 shows patients' adherence to the follow-up examinations conducted in our department of urogynecology. At times 4 (4/10) patients had completed the one-year follow-up, whereas the average follow-up for all patients was 7.4 months.

### 3.1. Complications

Postoperative complications were carefully analyzed and are listed in Table 7. We classified these complications by using the Clavien-Dindo grading of surgical complications [15, 16].

Furthermore, we divided the complications into early, midterm, and late complication according to the time of their occurrence.

**Figure 6:**
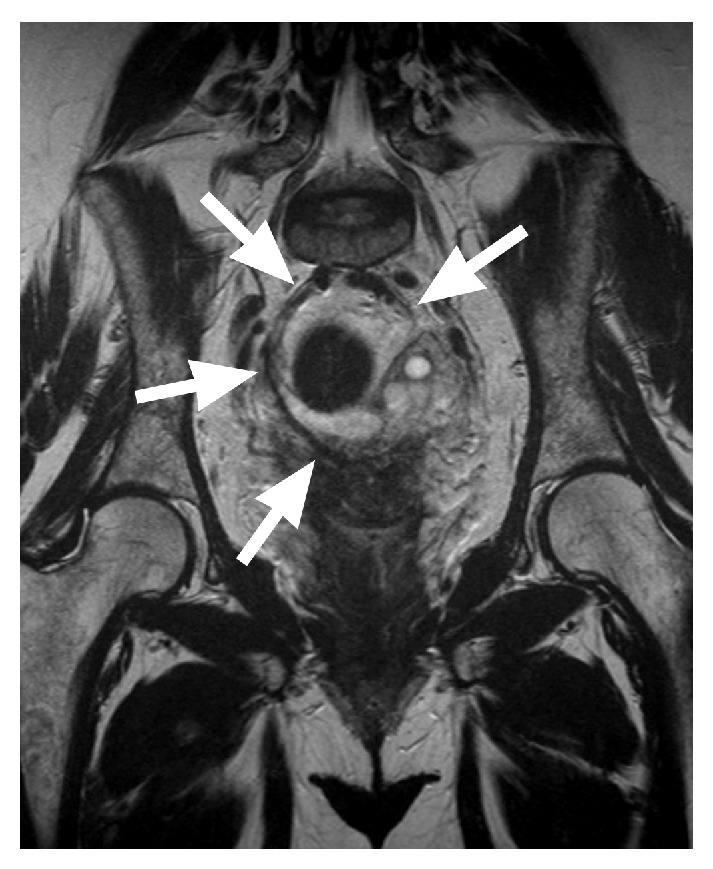
Coronal subvolume minimum intensity projection of a T2-weighted dataset, displaying the implant with a low signal intensity (arrows), comparable to the signal of muscle tissue.

**Figure 7:**
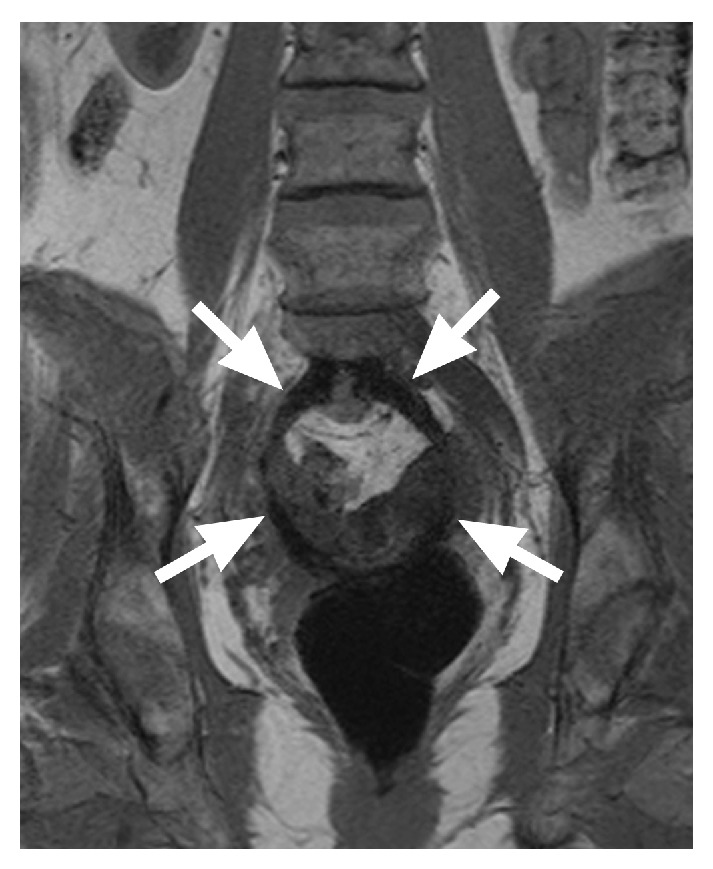
Same patient as in Figure 6, this time a T1-weighted dataset, again with a coronal subvolume minimum intensity projection. Using the T1-weighted images, the contrast between the implant and the surrounding tissue is even better. Due to the iron oxide particles, a signal loss in the area of the implant is obvious (arrows), which allows for exact identification of the implant.

Regarding early complications, one (1/10) patient suffered from paresthesia of the right thigh. We performed a MRI that revealed the implanted mesh in the desired lay and ruled out any neural compression or hematoma (Figures 6 and 7).

The symptoms declined after reassurance and use of NSAID for a few days and had completely resolved 1 month postoperatively. The same patient suffered from de-novo SUI, which required a placement of a TVT. Since the procedure was performed under regional anesthesia the patient was classified as suffering a complication grade III_a_.

Furthermore, 1 (1/10) patient suffered from persisting SUI, which required a placement of a TVT and 2 (2/10) patients reported mild pain in the sacral region, which was treated by NSAID in a low dose on demand for 2-3 weeks. There was no need for further intervention as the pain resolved in less than 3 weeks.

One (1/10) patient required a relaparoscopy on the second day postoperatively due to a hematoma of the right pelvic wall. The bleeding showed to be from the right ovarian vein and management required a laparoscopic salpingooophorectomy. This patient additionally had a recurrent UTI that was treated with an antibiotic. Since management of this patient required general anesthesia the patient was classified as suffering a complication grade III_b_.

There were no midterm complications whereas follow-up revealed one late complication comprising lower abdominal pain. Diagnostic laparoscopy revealed a 1.5 cm long opening in the peritoneum overlying the right lateral mesh arm approximately 2 cm from the cervical attachment point. Laparoscopic mobilizing of the peritoneum and closure above the underlying portion of the mesh were performed.

During the whole follow-up no vaginal mesh erosion, no chronic pelvic pain and no dyspareunia were seen. Apical success was observed in all the 10 (100%) patients and persisted throughout follow-up.

No recurrent prolapse surgery had to be performed. Only 1 (1/10) patient had a recurrence in the anterior compartment at 13 months follow-up showing a mild cystocele without any discomfort. Since the patient had normal bladder function and no relevant residual volume, no correction was indicated.

Thus to summarize the early complications we say that one patient had a complication classified as Clavien-Dindo grade I (UTI) and another one classified as Clavien-Dindo grade III_b_ (hematoma).

Another patient had one complication classified as Clavien-Dindo grade I (spontaneous resolving paresthesia of the thigh) and another one classified as Clavien-Dindo grade III_a_ (de-novo SUI). A third patient had a complication classified as Clavien-Dindo grade III_a_ (persistent SUI).

That means that we had early complications requiring a higher pharmacologic, surgical, or radiologic intervention (Clavien-Dindo grades higher than grade I) in 3 (3/10) patients, representing the relevant early complication rate.

## 4. Discussion

The uterosacral and cardinal ligaments (CL) are regarded the main anatomical support of the uterus and vault [17]. In a MRI-based study DeLancey estimated the lines of action and the tension load of both USL and CL showing that the tension on these ligaments is affected by their orientations [18].

As for the anatomical lay and histologic composition the USL can be divided into a superficial (USL_s_) and deep (USL_d_) part. The superficial part mainly comprises smooth muscle and connective tissue, whereas the deep part is of a neurovascular composition, as is the CL.

Taking this in regard, the USL_s_ seem to be the best accessible and anatomically safest part of both ligaments to operate on. Thus making it the most suitable to perform a site specific prolapse repair upon.

Regarding the operative technique, the critical steps are the dissection of a tunnel along the USL_s_ and the dissection at the sacrum at the level of S2.

The dissection of the tunnel has to be strictly subperitoneally. In the middle part of the uterosacral ligament (and the dissected canal) the distance to the ureter is 1–1.5 cm [19, 20]. Furthermore attention is paid to perform strict superficial preparation in order to keep a safe distance to the USL_d_ which is the neurovascular part.

Due to limited access when using a rigid laparoscopic instrument to create a curved tunnel of 5-6 cm of length, overdue tension at the peritoneum during dissection may accidently be applied. This may eventually cause a localized thinning of it. In the case of the patient who presented with a late complication of lower abdominal pain and localized opening in the peritoneum overlying part of the mesh (mentioned under 3.1 complications) this thinning may be a predisposing factor.

The complication of postoperative hematoma in the cervical region (mentioned under 3.1 complications) may be due to the supracervical hysterectomy. The later performed salpingooophorectomy was conducted because of a necrosis of the right ovary that may have been caused by a vascular shortage after hysterectomy or by electrocoagulation during hematoma revision.

Regarding the two patients who required a TVT, one had persistent SUI which means that 5/6 patients who suffered from SUI were cured after anatomical correction of the prolapse alone. On the other hand there was only 1/10 patients presenting with de-novo SUI. Due to the small sample size it is not possible to compare the apparently good results to other series, but as for this small sample results it seemed to be better than in a lot of published series [9].

This may also be related to our therapeutic protocol, since we only perform a colporrhaphy if the residual cysto- or rectocele after apical stabilization corresponds to POP-Q stage ≥ 2 (i.e., Aa, Ba, Ap, or Bp ≥ −1) to prevent undue overcorrection. In this series 1/10 patients had received a concomitant anterior and posterior colporrhaphy. Recently Leclaire found that a greater reduction in point Aa is a risk factor for de-novo SUI after sacropexy [9].

In terms of reduction of urge symptoms, we had 7/10 patients in whom OAB-dry dissolved as well as 1/10 with OAB-wet. Altogether 8/10 patients were relieved from OAB symptoms after the operation which is a good result. These results seem consistent with the results shown by abdominal mesh placement displayed in a series [21].

Summarizing, we had a 100% apical healing rate in this small sample of patients along with good anatomical results in the anterior compartment. The functional outcome seems to be favorable, yet a comparison with available data from abdominal or laparoscopic sacropexy series is not possible due to the small sample size.

In the ongoing debate on the use of meshes in prolapse surgery the choice of the material plays a critical role. The mesh we used is one with full ce-mark made up of PVDF. Many data suggest that this material has favorable properties. Comparison of PVDF and polypropylene (PP) in rodent model showed a better biocompatibility and less foreign body reaction with PVDF [22].

Furthermore it is well accepted that meshes for POP surgery should be macroporous. The used alloplastic material possesses a higher porosity under strain than most meshes do [23]. Further data are required to evaluate to what extent these properties may positively influence the postoperative outcome in patients.

The application of meshes made of PVDF is widely used in hernia repair. Berger and Bientzle reported a large prospective study in 2009 [24]. As for the application in POP-surgery, Noé et al. reported the use of this material in a prospective clinical trial [25].

The same mesh we applied was used for an abdominal procedure of abdominal sacropexy described by Jäger et al. who reported a cure rate of urge incontinence of 77% [21].

Regarding our knowledge about mesh properties after implantation it has to be stated that it is very limited in the case of sacropexy. Since the standard meshes are “invisible” for radiologic examinations or MRI the only way to evaluate them after implantation is by means of ultrasound.

Performing pelvic floor ultrasound provides valuable information about vaginal meshes but rather few information about meshes used for sacropexy because it is not capable of showing the meshes lying above the pelvis. Thus the relationship of the mesh to the sacral fixation point could not be investigated well so far.

Further on, a possible complication—like mesh detachment or compression of nerves or vessels in the presacral space—occurring above the pelvis could not be investigated through imaging so far and often required reoperation to visualize the area of concern.

In a step toward higher patients' safety effort was undertaken to enhance the visibility of meshes in radiologic examinations.

Krämer et al. first introduced a concept for MRI visualization of surgical meshes by integrating iron oxide particles into them in 2010 [14]. The first MRI visualization of an implanted mesh for inguinal hernia repair was reported in 2013 [26].

In our series we had three patients in whom we performed a MRI because of postoperative complaints. In these presented cases there was no need for surgical intervention since a complication was ruled out through MRI.

So the use of MRI comprises the only nonoperative way to visualize and evaluate the lay of an implanted mesh and may reduce the need for reoperations in case of postoperative complications. Additionally it gives the unique opportunity for the evaluation of changing mesh characteristics over time giving us new opportunities to study mesh behavior after implantation. This may be helpful in better understanding of causes for mesh related complications in POP surgery.

In this paper we presented the first in-human magnetic resonance visualization of a prolapse mesh implant. The performed MRIs showed a very good visualization of the mesh in addition to the nearby structures and were helpful in the management of postoperative complications.

## 5. Conclusion

Modified laparoscopic bilateral sacropexy (MLBS) is a feasible and safe operative procedure. The preliminary data are encouraging, showing favorable anatomical and functional outcome and good concomitant healing rates of SUI and OAB. The MRI-visibility of the implanted mesh has a good quality and is helpful in postoperative complication management. For further evaluation we are planning to perform a prospective study with a larger sample.

## Figures and Tables

**Table 1:** Pre-operative/baseline characteristics.

Age (yrs., mean)	62
Body mass index (kg/m^2^, mean)	25.7
Parity (*n*)	
1	2/10
2	2/10
≥3	6/10
Mode of delivery (*n*)	
Spontaneous	10/10
C. section/Forceps	0/10
Other obstetric risk factors (*n*)	
Birth weight > 4,000 gr.	0/10
Birth weight > 4,500 gr.	2/10
Perineal tear grades III or IV	1/10
Menopausal status (*n*)	
Premenopausal	2/10
Postmenopausal	8/10
Hormone replacement therapy (*n*)	3/8
History of prolapse surgery (*n*)	
Anterior compartment	3/10^∗^
Middle compartment	0/10
Posterior compartment	1/10^∗∗^
History of hysterectomy (*n*)	
Vaginal	3/10
Abdominal	0
Laparoscopic	0

^∗^One of these three patients has had an anterior colporrhaphy twice in her past medical history.

^∗∗^This patient has had a posterior colporrhaphy twice in her past medical history.

**Table 2:** Pre- and postoperative quantification of the prolapse.

	POP-Q measurements (cm)					
	Aa	Ba	C	D	Ap	Bp
Preoperative						
Mean	1.2	2.1	−0.2	−1.5	−2.1	−2.1
Median	1.0	1.5	−0.5	−2.0	−2.5	−2.5
Range	0 to +3	0 to +5	−4 to +3	−4 to +1	−3 to +1	−3 to +1
*n*	*10 *	*10 *	*10 *	*7* ^*^	*10 *	*10 *
Postoperative						
Mean	−1.5	−1.1	−6.3	−8.0	−2.7	−2.7
Median	−1.0	−1.0	−6.0	−8.0	−3.0	−3.0
Range	−3 to 0	−3 to 0	−7 to −5	−7 to −9	−3 to −1	−3 to −1
*n*	*10 *	*10 *	*10 *	*7* ^*^	*10 *	*10 *
Diff. postoperative to preoperative						
Mean	2.7	3.2	6.1	6.5	0.6	0.6
Median	2	2.5	5.5	6	0.5	0.5
Range	1 to 4	1 to 6	3 to 10	4 to 10	0 to 3	0–3
*n*	*10 *	*10 *	*10 *	*7* ^*^	*10 *	*10 *

^**∗**^3 patients had a hysterectomy in their past medical history, so that measurement D is not applicable.

**Table 3:** Pre- and postoperative quantification of the prolapse in respect of each compartment.

POP-Q measurement (cm) and POP-Q stage according to each compartment	Ant. compartment	Mid. compartment	Post. compartment
Preoperative			
Mean	Aa: +1.2/Ba: +2.1	C: −0.2/D: −1.5	Ap: −2.1/Bp: −2.1
Mean	Stage III	Stage II	Stage I
Median	Stage III	Stage II	Stage I
*n*	*10 *	*10 *	*10 *
Postoperative			
Mean	Aa: −1.5/Ba: −1.1	C: −6.3/D: −8.0	Ap: −2.7/Bp: −2.7
Mean	Stage I	Stage 0	Stage 0
Median	Stage I	Stage 0	Stage 0
*n*	*10 *	*10 *	*10 *

**Table 4:** Pre- and postoperative quantification of the urinary incontinence.

Incontinence	OAB-dry	OAB-wet	SUI	SUI grade		Frequency of mictur. *n*./d.^∗^	Nocturia *n*./d.^∗^	Pads used *n*./d.^∗^
				1	2			
Preoperative	9/10 (90%)	1/10 (10%)	6/10 (60%)	3/10 (30%)	3/10 (30%)	13.3	2.3	2
*n*:	10	10	10	10		10	10	8

Postoperative	2/10^∗∗^ (10%)	0/10 (0%)	2/10 (20%)	0/10 (0%)	2/10 (20%)	8.6	1.2	0.4
*n*:	10	10	10	10		8	8	7

^*^ *n./*d.: number per day.

^∗∗^One other patient had no evidence of OAB in the urodynamic or micturition diary and still reported urgency.

**Table 5:** Intra- and perioperative data.

Concomitant surgery (*n*)	
LASH (laparoscopic supracervical hysterectomy)	7/10
Salpingectomy	2/10
Salpingooophorectomy	5/10
Ovarian cystectomy	1/10
Adhesiolysis of omentum or bowel	5/10
Anterior colporrhaphy	1/10
Posterior colporrhaphy	1/10
Haemoglobin (mean, g/dL)	
Preoperative	13.9
Postoperative day	12.0
Postoperative before discharge	12.4
Need for analgesics postoperatively (*n*, %)	
Piritramid 0–6 h.	9/10
Piritramid or other short acting opioids 6–48 h.^∗^	1/10
NSAID in medium dose 6–48 h.	7/10
NSAID in low dose 6–48 h.	3/10
NSAID regularly in a low dose 3–5 d.	2/10
NSAID on demand in a low dose 3–5 d.	6/10
NSAID on demand in a low dose beyond 6 d.^∗∗^	1/10
Hospital stay (days)	
Mean	5.9
Range	3 to 11

^∗^Given in the intermediate care unit (ICU).

^∗∗^This patient was discharged and took NSAID on demand at home.

**Table 6:** Follow-up examinations.

	Follow-up 1	Follow-up 2	Follow-up 3	Follow-up 4	Follow-up
	3–6 weeks	2–4 months	6-7 months	11–14 months	(mean/range)
Follow-up completed (*n*)	1/10	9/10	6/10	4/10	7.4 months/1 to 14 months

**Table 7:** Description and classification of postoperative complications according to the Clavien-Dindo grading system.

Post-operative complications	Type of complication (*n*)	Management	Classification
			Clavien-Dindo grading sys.
Early complications days 01 to 30	Intraperitoneal hematoma^∗^	Relaparoscopy day 2	Grade III_b_
	1/10		1/10
	Recurrent UTI^∗^	Antibiotics	Grade I
	1/10		1/10
	Paraesthesia in right thigh^∗∗^	MRI/spontaneous resolving	Grade I
	1/10		1/10
	De-novo SUI^∗∗^	Urodynamics/TVT	Grade III_a_
	1/10		1/10
	Persistent SUI	Urodynamics/TVT	Grade III_a_
	1/10		1/10
	Mild sacral pain	Reassurance/NSAID	Grade I
	2/10		2/10

Midterm complications days 31 to 90	None		

Late complications >90 days	Lower abdominal pain (erosion of the peritoneum)	Relaparoscopy day 119	Grade III_b_
	1/10		1/10
	Recurrence of a mild cystocele	Not bothersome, no treatment.	Grade I
			1/10
	Intraperitoneal Hematoma^∗^ and adhesions.	Relaparoscopy	Grade III_b_
	1/10		1/10

^∗^The same patient in 3 occasions: 2 early and 1 late complications.

^∗∗^The same patient in 2 occasions: 2 early complications.

## Abbreviations

CL: Cardinal ligaments
FDA: Food and Drug Administration
ICS POP-Q: International Continence Society Pelvic Organ Prolapse Quantification
IHP: Inferior hypogastric plexus
MLBS: Modified laparoscopic bilateral sacropexy
MRI: Magnetic resonance imaging
NSAID: Nonsteroidal anti-inflammatory drug
OAB: Overactive bladder
POP: Pelvic organ prolapse
PP: Polypropylene
PVDF: Polyvinylidene fluoride
SUI: Stress urinary incontinence
S2: Second sacral vertebrae
TVT: Tension-free vaginal tape
USL: Uterosacral ligaments
USL_s_: Superficial portion of the uterosacral ligament
USL_d_: Deep portion of the uterosacral ligament
UTI: Urinary tract infection.
